# Distribution of Variables by Method of Outlier Detection

**DOI:** 10.3389/fpsyg.2012.00211

**Published:** 2012-07-05

**Authors:** W. Holmes Finch

**Affiliations:** ^1^Department of Educational Psychology, Ball State UniversityMuncie, IN, USA

**Keywords:** Mahalanobis distance, minimum covariance determinant, minimum generalized variance, minimum volume ellipsoid, outliers, projection

## Abstract

The presence of outliers can very problematic in data analysis, leading statisticians to develop a wide variety of methods for identifying them in both the univariate and multivariate contexts. In case of the latter, perhaps the most popular approach has been Mahalanobis distance, where large values suggest an observation that is unusual as compared to the center of the data. However, researchers have identified problems with the application of this metric such that its utility may be limited in some situations. As a consequence, other methods for detecting outlying observations have been developed and studied. However, a number of these approaches, while apparently robust and useful have not made their way into general practice in the social sciences. Thus, the goal of this study was to describe some of these methods and demonstrate them using a well known dataset from a popular multivariate textbook widely used in the social sciences. Results demonstrated that the methods do indeed result in datasets with very different distributional characteristics. These results are discussed in light of how they might be used by researchers and practitioners.

## Introduction

The presence of outliers is a ubiquitous and sometimes problematic aspect of data analysis. They can result from a variety of processes, including data recording and entry errors, obtaining samples from other than the target population, and sampling unusual individuals from the target population itself (Kruskal, 1988). Based on the standard definition of outliers, it is entirely possible that a dataset may not have any such cases, or it might have many. Given that they can arise from very different processes, outliers should not all be treated in the same manner. For example, those caused by data collection problems are likely to be removed from the sample prior to analysis, while those that are simply unusual members of the target population would be retained for data analysis. Finally, Kruskal noted that in some cases understanding the mechanism that caused outliers is the most important aspect of a given study. In other words, outliers can themselves provide useful information to researchers, and are not necessarily problematic in the sense of being bad data. The focus of this manuscript is not on the mechanism giving rise to outliers, but rather on methods for detecting them once they are present in the sample.

A number of authors have sought to precisely define what constitutes an outlier (e.g., Evans, 1999), and methods for detecting and dealing with them once detected remain an active area of research. It is well known that outliers can have a dramatic impact on the performance of common statistical analyses such as Pearson’s correlation coefficient (Marascuilo and Serlin, 1988), univariate, and multivariate means comparisons (Kirk, 1995; Huberty and Olejnik, 2006), cluster analysis (Kaufman and Rousseeuw, 2005), multivariate means comparisons, and factor analysis (Brown, 2006), among others. For this reason researchers are strongly encouraged to investigate their data for the presence of outliers prior to conducting data analysis (Tabachnick and Fidell, 2007).

In the multivariate context, the most commonly recommended approach for outlier detection is the Mahalanobis Distance (*D*^2^). While this approach can be an effective tool for such purpose, it also has weaknesses that might render it less than effective in many circumstances (Wilcox, 2005). The focus of this manuscript is on describing several alternative methods for multivariate outlier detection; i.e., observations that have unusual patterns on multiple variables as opposed to extreme scores on a single variable (univariate outliers). In addition, these approaches will be demonstrated, along with *D*^2^, using a set of data taken from Tabachnick and Fidell (2007). The demonstration will utilize functions from the R software package for both outlier detection and data analysis after removal of the outliers. It should be noted that the focus of this manuscript is not on attempting to identify some optimal approach for dealing with outliers once they have been identified, which is an area of statistics itself replete with research, and which is well beyond the scope of this study. Suffice it to say that identification of outliers is only the first step in the process, and much thought must be given to how outliers will be handled. In the current study, they will be removed from the dataset in order to clearly demonstrate the differential impact of the various outlier detection methods on the data and subsequent analyses. However, it is not recommended that this approach to outliers be taken in every situation.

### Impact of outliers in multivariate analysis

Outliers can have a dramatic impact on the results of common multivariate statistical analyses. For example, they can distort correlation coefficients (Marascuilo and Serlin, 1988; Osborne and Overbay, 2004), and create problems in regression analysis, even leading to the presence of collinearity among the set of predictor variables in multiple regression (Pedhazur, 1997). Distortions to the correlation may in turn lead to biased sample estimates, as outliers artificially impact the degree of linearity present between a pair of variables (Osborne and Overbay, 2004). In addition, methods based on the correlation coefficient such as factor analysis and structural equation modeling are also negatively impacted by the presence of outliers in data (Brown, 2006). Cluster analysis is particularly sensitive to outliers with a distortion of cluster results when outliers are the center or starting point of the analysis (Kaufman and Rousseeuw, 2005). Outliers can also themselves form a cluster, which is not truly representative of the broader array of values in the population. Outliers have also been shown to detrimentally impact testing for mean differences using ANOVA through biasing group means where they are present (Osborne and Overbay, 2004).

While outliers can be problematic from a statistical perspective, it is not always advisable to remove them from the data. When these observations are members of the target population, their presence in the dataset can be quite informative regarding the nature of the population (e.g., Mourão-Miranda et al., 2011). To remove outliers from the sample in this case would lead to loss of information about the population at large. In such situations, outlier detection would be helpful in terms of identifying members of the target population who are unusual when compared to the rest, but these individuals should not be removed from the sample (Zijlstra et al., 2011).

### Methods of multivariate outlier detection

Given the negative impact that outliers can have on multivariate statistical methods, their accurate detection is an important matter to consider prior to data analysis (Tabachnick and Fidell, 2007; Stevens, 2009). In popular multivariate statistics texts, the reader is recommended to use *D*^2^ for multivariate outlier detection, although as is described below, there are several alternatives for multivariate outlier detection that may prove to be more effective than this standard approach. Prior to discussing these methods however, it is important to briefly discuss general qualities that make for an effective outlier detection method. Readers interested in a more detailed treatment are referred to two excellent texts by Wilcox (2005, 2010).

When thinking about the impact of outliers, perhaps the key consideration is the breakdown point of the statistical analysis in question. The breakdown point can be thought of as the minimum proportion of a sample that can consist of outliers after which point they will have a notable impact on the statistic of interest. In other words, if a statistic has a breakdown point of 0.1, then 10% of the sample could consist of outliers without markedly impacting the statistic. However, if the next observation beyond this 10% was also an outlier, the statistic in question would then be impacted by its presence (Maronna et al., 2006). Comparatively, a statistic with a breakdown point of 0.3 would be relatively more impervious to outliers, as it would not be impacted until more than 30% of the sample was made up of outliers. Of course, it should be remembered that the degree of this impact is dependent on the magnitude of the outlying observation, such that more extreme outliers would have a greater impact on the statistic than would a less extreme value. A high breakdown point is generally considered to be a positive attribute.

While the breakdown point is typically thought of as a characteristic of a statistic, it can also be a characteristic of a statistic in conjunction with a particular method of outlier detection. Thus, if a researcher calculates the sample mean after removing outliers using a method such as *D*^2^, the breakdown point of the combination of mean and outlier detection method will be different than that of the mean by itself. Finally, although having a high breakdown point is generally desirable, it is also true that statistics with higher breakdown points (e.g., the median, the trimmed mean) are often less accurate in estimating population parameters when the data are drawn from a multivariate normal distribution (Genton and Lucas, 2003).

Another important property for a statistical measure of location (e.g., mean) is that it exhibit both location and scale equivariance (Wilcox, 2005). Location equivariance means that if a constant is added to each observation in the data set, the measure of location will be increased by that constant value. Scale equivariance occurs when multiplication of each observation in the data set by a constant leads to a change in the measure of location by the same constant. In other words, the scale of measurement should not influence relative comparisons of individuals within the sample or relative comparisons of group measures of location such as the mean. In the context of multivariate data, these properties for measures of location are referred to as affine equivariance. Affine equivariance extends the notion of equivariance beyond changes in location and scale to measures of multivariate dispersion. Covariance matrices are affine equivariant, for example, though they are not particularly robust to the presence of outliers (Wilcox, 2005). A viable approach to dealing with multivariate outliers must maintain affine equivariance.

Following is a description of several approaches for outlier detection. For the most part, these descriptions are presented conceptually, including technical details only when they are vital to understanding how the methods work. References are provided for the reader who is interested in learning more about the technical aspects of these approaches. In addition to these descriptions, Table 1 also includes a summary of each method, including fundamental equations, pertinent references, and strengths and weaknesses.

**Table 1:** **Summary of outlier detection methods**.

Method	Equation	Reference	Strengths	Weaknesses
$D_{i}^{2}$	${\left(x_{i}-\bar{x}\right)}^{\prime }S^{-1}\left(x_{i}-\bar{x}\right)$	Mahalanobis (1936)	Intuitively easy to understand; easy to calculate; familiar to other researchers	Sensitive to outliers; assumes data are continuous
MVE	Identify subset of data contained within the ellipsoid that has minimized volume	Rousseeuw and Leroy (1987)	Yields mean with maximum possible breakdown point	May remove as much as 50% of sample
MCD	Identify subset of data that minimizes the determinant of the covariance matrix	Rousseeuw and van Driessen (1999)	Yields mean with maximum possible breakdown point	May remove as much as 50% of sample
MGV	Calculate MAD version of *D*^2^ as $\sum_{j=1}^{n}\sqrt{\sum_{l=1}^{p}{\left(\frac{x_{jl}-x_{il}}{\text{MA}{\text{D}}_{l}}\right)}^{2}}$ to identify most central points; calculate variance of this central set as additional observations are added one by one; examine this generalized variance and retain those with values less than the adjusted median $M_{\text{G}}+\sqrt{\chi_{0.975.p}^{2}}\left(q_{\text{3}}-q_{\text{1}}\right)$	Wilcox (2005)	Typically removes fewer observations than either MVE or MCD	Generally does not have as high a breakdown point as MVE or MCD
P1	Identify the multivariate center of data using MCD or MVE and then determine its relative distance from this center (depth); use the MGV criteria based on this depth to identify outliers	Donoho and Gasko (1992)	Approximates an affine equivariant outlier detection method; may not exclude as many cases as MVE or MCD	Will not typically lead to a mean with the maximum possible breakdown point
P2	Identify all possible lines between all pairs of observations in order to determine depth of each point	Donoho and Gasko (1992)	Some evidence that this method is more accurate than P1 in terms of identifying outliers	Extensive computational time, particularly for large datasets
P3	Same approach as P1 except that the criteria for identifying outliers is $M_{\text{D}}+\sqrt{\chi_{0.975.p}^{2}}\left(\frac{\text{MA}{\text{D}}_{i}}{0.6745}\right)$	Donoho and Gasko (1992)	May yield a mean with a higher breakdown point than other projection methods	Will likely lead to exclusion of more observations as outliers than will other projection approaches

### Mahalanobis distance

The most commonly recommended approach for multivariate outlier detection is *D*^2^, which is based on a measure of multivariate distance first introduced by Mahalanobis (1936), and which has been used in a wide variety of contexts. *D*^2^ has been suggested as the default outlier detection method by a number of authors in popular textbooks used by researchers in a variety of fields (e.g., Johnson and Wichern, 2002; Tabachnick and Fidell, 2007). In practice, a researcher would first calculate the value of *D*^2^ for each subject in the sample, as follows:

(1) $$D_{i}^{2}={\left(x_{i}-\bar{x}\right)}^{\prime }S^{-1}\left(x_{i}-\bar{x}\right)$$

where

*x_i_* = Vector of scores on the set of *p* variables for subject *i*
$\bar{x}$ = Vector of sample means on the set of *p* variables
*S* = Covariance matrix for the *p* variables

A number of recommendations exist in the literature for identifying when this value is large; i.e., when an observation might be an outlier. The approach used here will be to compare $D_{i}^{2}$ to the χ^2^ distribution with *p* degrees of freedom and declare an observation to be an outlier if its value exceeds the quantile for some inverse probability; i.e., $\chi_{p}^{2}\left(0.005\right)$ (Mahalanobis).

*D*^2^ is easy to compute using existing software and allows for direct hypothesis testing regarding outlier status (Wilcox, 2005). Despite these advantages, *D*^2^ is sensitive to outliers because it is based on the sample covariance matrix, ***S***, which is itself sensitive to outliers (Wilcox, 2005). In addition, *D*^2^ assumes that the data are continuous and not categorical so that when data are ordinal, for example, it may be inappropriate for outlier detection (Zijlstra et al., 2007). Given these problems, researchers have developed alternatives to multivariate outlier detection that are more robust and more flexible than *D*^2^.

### Minimum volume ellipsoid

**Figure 1:**
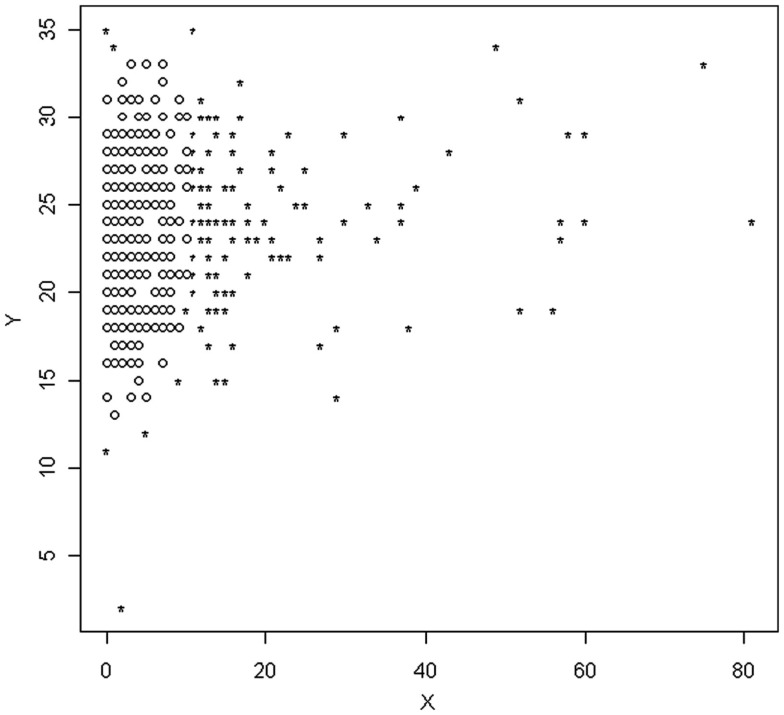
**Scatterplot of observations identified as outliers based on the MVE method**.

One of the earliest of alternative approach to outlier detection was the Minimum Volume Ellipsoid (MVE), developed by Rousseeuw and Leroy (1987). In concept, the goal behind this method is to identify a subsample of observations of size *h* (where *h* < *n*) that creates the smallest volume ellipsoid of data points, based on the values of the variables. By definition, this ellipsoid should be free of outliers, and estimates of central tendency and dispersion would be obtained using just this subset of observations. The MVE approach to dealing with outliers can, in practice, be all but intractable to carry out as the number of possible ellipsoids to investigate will typically be quite large. Therefore, an alternative approach is to take a large number of random samples of size *h* with replacement, where

(2) $$h=\frac{n}{2}+1,$$

and calculate the volume of the ellipsoids created by each. The final sample to be used in further analyses is that which yields the smallest ellipsoid. An example of such an ellipsoid based on MVE can be seen in Figure 1. The circles represent observations that have been retained, while those marked with a star represent outliers that will be removed from the sample for future analyses.

### Minimum covariance determinant

The minimum covariance determinant (MCD) approach to outlier detection is similar to the MVE in that it searches for a portion of the data that eliminates the presence and impact of outliers. However, whereas MVE seeks to do this by minimizing the volume of an ellipsoid created by the retained points, MCD does it by minimizing the determinant of the covariance matrix, which is an estimate of the generalized variance in a multivariate set of data (Rousseeuw and van Driessen, 1999). The dataset with the smallest determinant will be the one least influenced by outliers and which can then be used for future statistical analyses. Statistics calculated on data to which MCD and MVE have been applied will typically have high breakdown points (Rousseeuw and van Driessen, 1999).

**Figure 2:**
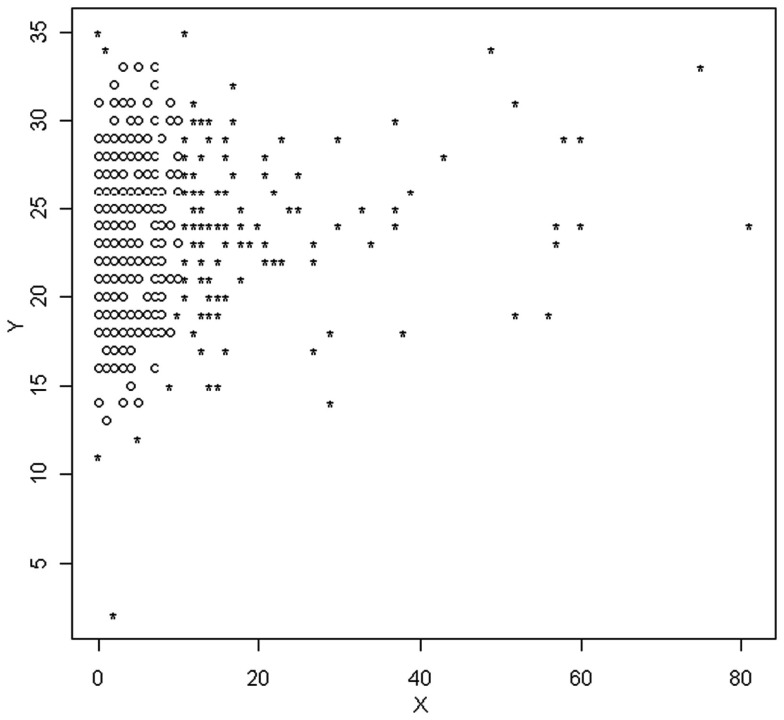
**Scatterplot of observations identified as outliers based on the MCD method**.

As with MVE, the logistics of searching every possible subset of the data of size *h* to find the one that yields the smallest determinant are not practical in the vast majority of situations. As a consequence Rousseeuw and van Driessen (1999) developed a multiple step algorithm to approximate the MCD, obviating the need to examine all possible subsets of the data. This approach, known as Fast MCD involves the random selection of an initial subsample from the data of size *h*, for which the values of $D_{i}^{2}$ are calculated and ordered from smallest to largest. The *h* smallest $D_{i}^{2}$ values (and thus the data points associated with them) are then retained into a new subset of the data, after which individuals from the full dataset are randomly added and the value of the determinant calculated. The algorithm stops when it attains a subsample (size *h*) of the full data that yields the smallest determinant. Variants of this algorithm involve the selection of multiple subsamples in the initial step, and with several minimization procedures running parallel to one another simultaneously (Hardin and Rocke, 2004). Figure 2 includes a scatterplot identifying individuals as outliers based on the MCD method. Again, outliers are marked with a star.

### Minimum generalized variance

One potential difficulty with both MVE and MCD is that they tend to identify a relatively large number of outliers when the variables under examination are not independent of one another (Wilcox, 2005). A third approach for outlier detection that was designed to avoid this problem is the Minimum Generalized Variance (MGV). MGV is based on a similar principle to MCD in that the set of data with the smallest overall variance is identified. However, rather than relying on the random addition of observations to the core data set to be retained, it includes those individuals whose inclusion increases the generalized variance as little as possible.

As with MVE and MCD, MGV is an iterative procedure. In the first step the *p* most centrally located points are identified using a non-parametric estimate of *D_i_* which is calculated as

(3) $$D_{i}=\overset{n}{\underset{j=1}{\sum }}\sqrt{\overset{p}{\underset{l=1}{\sum }}{\left(\frac{x_{jl}-x_{il}}{\text{MA}{\text{D}}_{l}}\right)}^{2}},$$

where

(4) $${\text{MAD}}_{l}=\text{MED}\left\{\left[x_{i}-M\right]\right\}.$$

**Figure 3:**
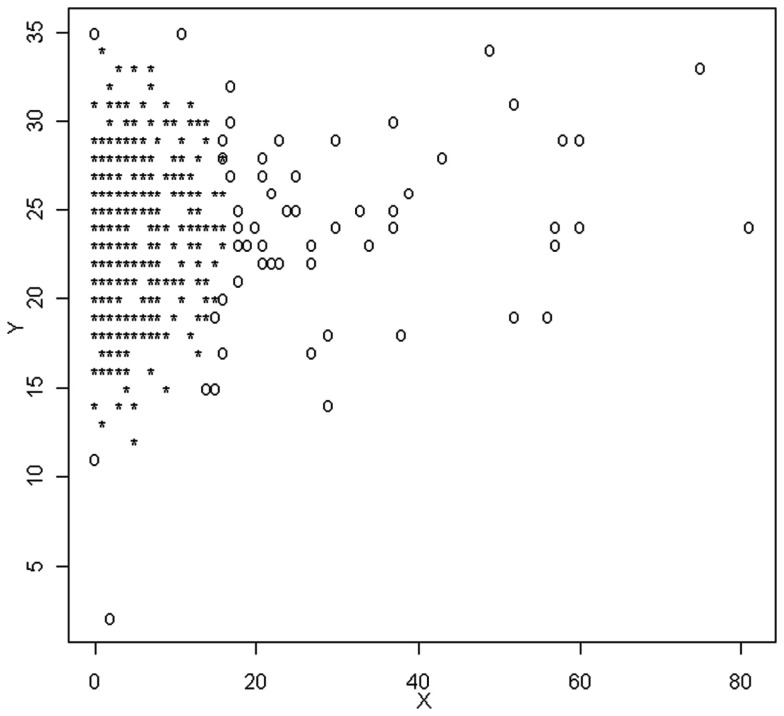
**Scatterplot of observations identified as outliers based on the MGV method**.

In other words, MAD, the median absolute deviation, is the median of the deviations between each individual data point and the median of the data set, *M*. The most centrally located observations are those with the smallest value of *D_i_* as calculated above. These points are then placed in a new data set, after which the generalized variance associated with adding each of the remaining observations not originally placed in this new data is calculated. The observation with the smallest generalized variance is then added to the new data set. For each data point remaining outside of the new data set, the generalized variance is recalculated, accounting for the new observation that was just added. Once again, the observation with the lowest generalized variance is then added to the new data set. This process is repeated until all of the original data points are included in the new data set; i.e., the new data set is identical in terms of membership to the old one. However, now each observation has associated with it a value for the generalized variance. Observations that are more distant from the bulk of the data will have larger values of the generalized variance. For *p *= 2 variables, observations with a generalized variance greater than

(5) $$q_{3}+1.5\left(q_{3}-q_{1}\right)$$

would be considered outliers, where *q*_1_ and *q*_2_ are the lower and upper quartiles, respectively, of the generalized variances. For more than two variables, the generalized variances are compared with

(6) $$M_{G}+\sqrt{\chi_{0.975.p}^{2}}\left(q_{3}-q_{1}\right),$$

where *M*_G_ is the median of the generalized variance values and $\chi_{0.975.p}^{2}.$ Figure 3 is a plot of *X* and *Y*, with outliers identified using the MGV approach. In this graph, outliers are denoted by 0, which is different than notation used in Figures 1 and 2. These graphs are included here exactly as taken from the R software output, which will be used extensively in the following examples.

### Projection-based outlier detection

Another alternative for identifying multivariate outliers is based on the notion of the depth of one data point among a set of other points. The idea of depth was described by Tukey (1975), and later expanded upon by Donoho and Gasko (1992). In general, depth can be thought of as the relative location of an observation vis-à-vis either edge (upper or lower) in a set of data. In the univariate case, this simply means determining to which edge a given observation more closely lies (i.e., maximum or minimum value), and then calculating the proportion of cases between that observation and its closest edge. The larger this proportion, the deeper the observation lies in the univariate data. While mathematically somewhat more challenging, conceptually projection methods of multivariate outlier detection work in much the same way. However, rather than determining the proximity to a single edge, the algorithm must identify the proximity to the edge of the multivariate space. This process is carried out using the method of projection that is described below.

For the purposes of this explanation, we will avoid presenting the mathematical equations that underlie the projection-based outlier detection approach. The interested reader is encouraged to refer to Wilcox (2005) for a more technical treatment of this methodology. Following is a conceptual description of two commonly used approaches to carrying out this technique when *p *= 2. In the first method, the algorithm begins by identifying the multivariate center of the data using an acceptable approach, such as the multivariate mean after application of MCD or MVE. Next, for each point (***X_i_***) the following steps are carried out:

(1) A line is drawn connecting the multivariate center and point ***X_i_***.
(2) A line perpendicular to the line in 1 is then drawn from each of the other observations, ***X_j_***.
(3) The location where the line in 2 intersects with the line in 1 is the projected depth (*d_ij_*) of that data point for the line.
(4) Steps 1–3 are then repeated such that each of the *n* data points is connected to the multivariate center of the data with a line, and corresponding values of *d_ij_* are calculated for each of the other observations.
(5) For a given observation, each of its depth values, *d_ij_*, is compared with a standard (to be described below). If for any single projection the observation is an outlier, then it is classified as an outlier for purposes of future analyses.

As mentioned earlier, there is an alternative approach to the projection method, which is not based on finding the multivariate center of the distribution. Rather, all $\left(n^{2}-n\right)/2$ possible lines are drawn between all pairs of observations in the dataset. Then, the approach outlined above for calculating *d_ij_* is used for each of these lines. Thus, rather than having *n−*1 such *d_ij_* values, each observation will have $\frac{\left(n^{2}-n\right)}{2}-1$ indicators of depth. In all other ways, this second approach is identical to the first, however. Prior research has demonstrated that this second method might be more accurate than the first, but it is not clear how great an advantage it actually has in practice (Wilcox, 2005). Furthermore, because it must examine all possible lines in the set of data, method 2 can require quite a bit more computational time, particularly for large datasets.

The literature on multivariate outlier detection using the projection-based method includes two different criteria against which an observation can be judged as an outlier. The first of these is essentially identical to that used for the MGV in Eq. 6, with the exception that *M*_G_ is replaced by *M_D_*, the median of the *d_ij_* for that projection. Observations associated with values of *d_ij_* larger than this cut score are considered to be outliers for that projection. An alternative comparison criterion is

(7) $$M_{\text{D}}+\sqrt{\chi_{0.975.p}^{2}}\left(\frac{{\text{MAD}}_{\text{i}}}{0.6745}\right)$$

where *MAD_i_* is the median of all |*d_ij_ − M_D_*|. Here, MAD*_i_* is scaled by the divisor 0.6745 so that it approximates the standard deviation obtained when sampling from a normal distribution. Regardless of the criterion used, an observation is declared to be an outlier in general if it is an outlier for any of the projections.

### Goals of the current study

The primary goal of this study was to describe alternatives to *D*^2^ for multivariate outlier detection. As noted above, *D*^2^ has a number of weaknesses in this regard, making it less than optimal for many research situations. Researchers need outlier detection methods on which they can depend, particularly given the sensitivity of many multivariate statistical techniques to the presence of outliers. Those described here may well fill that niche. A secondary goal of this study was to demonstrate, for a well known dataset, the impact of the various outlier detection methods on measures of location, variation and covariation. It is hoped that this study will serve as a useful reference for applied researchers working in the multivariate context who need to ascertain whether any observations are outliers, and if so which ones.

## Materials and Methods

The Women’s Health and Drug study that is described in detail in Tabachnick and Fidell (2007) was used for demonstrative purposes. This dataset was selected because it appears in this very popular text in order to demonstrate data screening and as such was deemed an excellent source for demonstrating the methods studied here. A subset of the variables were used in the study, including number of visits to a health care provider (TIMEDRS), attitudes toward medication (ATTDRUG) and attitudes toward housework (ATTHOUSE). These variables were selected because they were featured in Tabachnick and Fidell’s own analysis of the data. The sample used in this study consisted of 465 females aged 20–59 years who were randomly sampled from the San Fernando Valley in California and interviewed in 1976. Further description of the sample and the study from which it was drawn can be found in Tabachnick and Fidell.

In order to explore the impact of the various outlier detection methods included here, a variety of statistical analyses were conducted subsequent to the application of each approach. In particular, distributions of the three variables were examined for the datasets created by the various outlier detection methods, as well as the full dataset. The strategy in this study was to remove all observations that were identified as outliers by each method, thus creating datasets for each approach that included only those not deemed to be outliers. It is important to note that this is not typically recommended practice, nor is it being suggested here. Rather, the purpose of this study was to demonstrate the impact of each method on the data itself. Therefore, rather than take the approach of examining each outlier carefully to ascertain whether it was truly part of the target population, the strategy was to remove those cases identified as outliers prior to conducting statistical analyses. In this way, it was hoped that the reader could clearly see the way in which each detection method worked and how this might impact resulting analyses. In terms of the actual data analysis, the focus was on describing the resulting datasets. Therefore, distributional characteristics of each variable within each method were calculated, including the mean, median, standard deviation, skewness, kurtosis, and first and third quartiles. In addition, distributions of the variables were examined using the boxplot. Finally, in order to demonstrate the impact of these approaches on relational measures, Pearson’s correlation coefficient was estimated between each pair of variables. All statistical analyses including identification of outliers was carried out using the R software package, version 2.12.1 (R Foundation for Statistical Computing, 2010). The R code used to conduct these analyses appears in the Appendix at the end of the manuscript.

## Results

**Figure 4:**
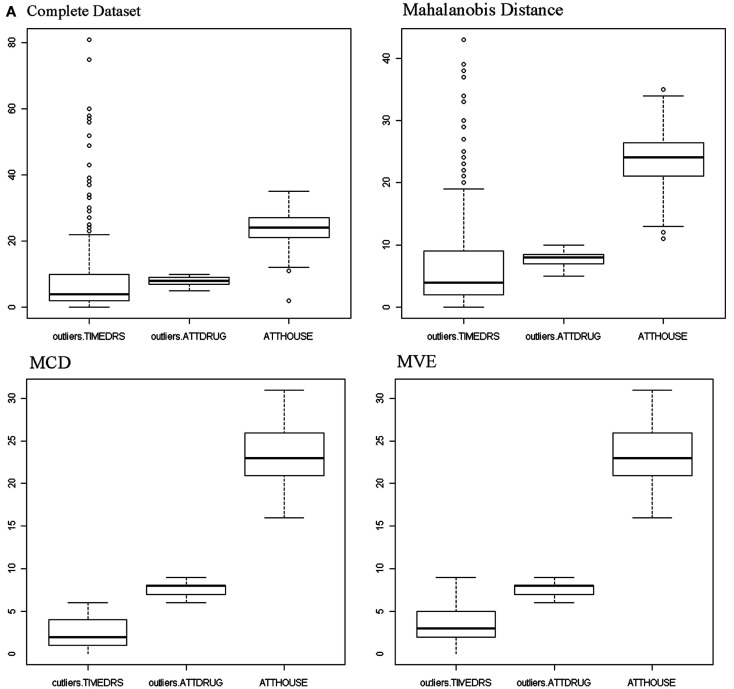

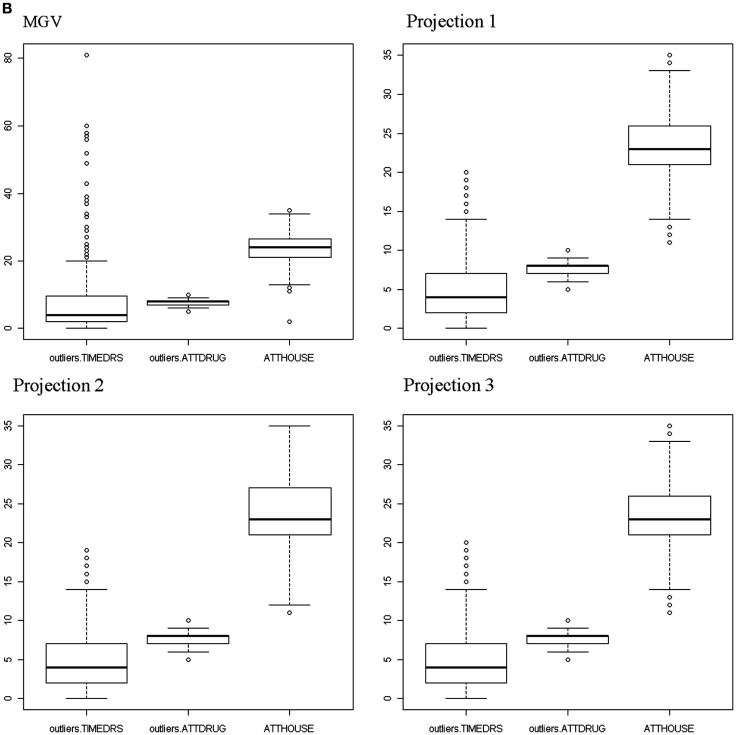
**Boxplots**.

An initial examination of the full dataset using boxplots appears in Figure 4. It is clear that in particular the variable TIMEDRS is positively skewed with a number of fairly large values, even while the median is well under 10. Descriptive statistics for the full dataset (Table 2) show that indeed, all of the variables are fairly kurtotic, particularly TIMEDRS, which also displays a strong positive skew. Finally, the correlations among the three variables for the full dataset appear in Table 3. All of these are below 0.15, indicating fairly weak relationships among the measures. However, it is not clear to what extent these correlations may be impacted by the distributional characteristics just described.

**Table 2:** **Descriptive statistics**.

Variable	Mean							
	Full (*N* = 465)	*D*^2^ (*N* = 452)	MCD (*N* = 235)	MVE (*N* = 235)	MGV (*N* = 463)	P1 (*N* = 425)	P2 (*N* = 422)	P3 (*N* = 425)
TIMEDRS	7.90	6.67	2.45	3.37	7.64	5.27	5.17	5.27
ATTDRUG	7.69	7.67	7.69	7.68	7.68	7.65	7.64	7.65
ATTHOUSE	23.53	23.56	23.36	23.57	23.50	23.54	23.54	23.54
**MEDIAN**								
TIMEDRS	4.00	4.00	2.00	3.00	2.00	2.00	2.00	2.00
ATTDRUG	8.00	8.00	8.00	8.00	7.00	7.00	7.00	7.00
ATTHOUSE	24.00	24.00	23.00	23.00	21.00	21.00	21.00	21.00
**Q1**								
TIMEDRS	2.00	2.00	1.00	2.00	2.00	2.00	2.00	2.00
ATTDRUG	7.00	7.00	7.00	7.00	7.00	7.00	7.00	7.00
ATTHOUSE	21.00	21.00	21.00	21.00	21.00	21.00	21.00	21.00
**Q3**								
TIMEDRS	10.00	9.00	4.00	5.00	9.50	7.00	7.00	7.00
ATTDRUG	9.00	8.25	8.00	8.00	8.00	8.00	8.00	8.00
ATTHOUSE	27.00	26.25	26.00	26.00	26.50	26.00	26.75	26.00
**STANDARD DEVIATION**								
TIMEDRS	10.95	7.35	1.59	2.22	10.23	4.72	4.58	4.72
ATTDRUG	1.16	1.16	0.89	0.81	1.15	1.56	1.52	1.56
ATTHOUSE	4.48	4.22	3.67	3.40	4.46	4.25	4.26	4.25
**SKEWNESS**								
TIMEDRS	3.23	2.07	0.23	0.46	3.15	1.12	1.08	1.12
ATTDRUG	−0.12	−0.11	−0.16	0.01	−0.12	−0.09	−0.09	−0.09
ATTHOUSE	−0.45	−0.06	−0.03	0.06	−0.46	−0.03	−0.03	−0.03
**KURTOSIS**								
TIMEDRS	15.88	7.92	2.32	2.49	15.77	3.42	3.29	3.42
ATTDRUG	2.53	2.51	2.43	2.31	2.54	2.51	2.51	2.51
ATTHOUSE	4.50	2.71	2.16	2.17	4.54	2.69	2.68	2.69

**Table 3:** **Correlations**.

Variable	TIMEDRS	ATTDRUG	ATTHOUSE
**FULL DATA SET (*N* = 465)**			
TIMEDRS	1.00	0.10	0.13
ATTDRUG	0.10	1.00	0.03
ATTHOUSE	0.13	0.03	1.00
**MAHALANOBIS DISTANCE (*N* = 452)**			
TIMEDRS	1.00	0.07	0.08
ATTDRUG	0.07	1.00	0.02
ATTHOUSE	0.08	0.02	1.00
**MCD (*N* = 235)**			
TIMEDRS	1.00	0.25	0.19
ATTDRUG	0.25	1.00	0.26
ATTHOUSE	0.19	0.26	1.00
**MVE (*N* = 235)**			
TIMEDRS	1.00	0.33	0.05
ATTDRUG	0.33	1.00	0.32
ATTHOUSE	0.05	0.32	1.00
**MGV (*N* = 463)**			
TIMEDRS	1.00	0.07	0.10
ATTDRUG	0.07	1.00	0.02
ATTHOUSE	0.10	0.02	1.00
**PROJECTION 1 (*N* = 425)**			
TIMEDRS	1.00	0.06	0.10
ATTDRUG	0.06	1.00	0.03
ATTHOUSE	0.10	0.03	1.00
**PROJECTION 2 (*N* = 422)**			
TIMEDRS	1.00	0.04	0.11
ATTDRUG	0.04	1.00	0.03
ATTHOUSE	0.11	0.03	1.00
**PROJECTION 3 (*N* = 425)**			
TIMEDRS	1.00	0.06	0.10
ATTDRUG	0.06	1.00	0.03
ATTHOUSE	0.10	0.03	1.00

Given these distributional issues, the researcher working with this dataset would be well advised to investigate the possibility that outliers are present. For this example, we can use R to calculate *D*^2^ for each observation, with appropriate program code appearing in the Appendix. In order to identify an observation as an outlier, we compare the *D*^2^ value to the chi-square distribution with degrees of freedom equal to the number of variables (three in this case), and α = 0.001. Using this criterion, 13 individuals were identified as outliers. In order to demonstrate the impact of using *D*^2^ for outlier detection, these individuals were removed, and descriptive graphics and statistics were generated for the remaining 452 observations. An examination of the boxplot for the Mahalanobis data reveals that the range of values is more truncated than for the original, particularly for TIMEDRS. A similar result is evident in the descriptive statistics found in Table 1, where we can see that the standard deviation, skewness, kurtosis and mean are all smaller in the Mahalanobis data for TIMEDRS. In contrast, the removal of the 13 outliers identified by *D*^2^ did not result in great changes for the distributional characteristics of ATTDRUG or ATTHOUSE. The correlations among the three variables were comparable to those in the full dataset, if not slightly smaller.

As discussed previously, there are some potential problems with using *D*^2^ as a method for outlier detection. For this reason, other approaches have been suggested for use in the context of multivariate data in particular. A number of these, including MCD, MVE, MGV, and three projection methods, were applied to this dataset, followed by generation of graphs and descriptive statistics as was done for both the full dataset and the Mahalanobis data. Boxplots for the three variables after outliers were identified and removed by each of the methods appear in Figure 4. As can be seen, the MCD and MVE approaches resulted in data that appear to be the least skewed, particularly for TIMEDRS. In contrast, the data from MGV was very similar to that of the full dataset, while the three projection methods resulted in data that appeared to lie between MCD/MVE and the Mahalanobis data in terms of the distributions of the three variables. An examination of Table 1 confirms that making use of the different outlier detection methods results in datasets with markedly different distributional characteristics. Of particular note are differences between MCD/MVE as compared to the full dataset, and the Mahalanobis and MGV data. Specifically, the skewness and kurtosis evident in these two samples was markedly lower than that of any of the datasets, particularly the full and MGV datasets. In addition, probably as a result of removing a number of individuals with large values, the mean of TIMEDRS was substantially lower for the MCD and MVE data than for the full, Mahalanobis, and MGV datasets. As noted with the boxplot, the three projection methods produced means, skewness, and kurtosis values that generally fell between those of MCD/MVE and the other approaches. Also of interest in this regard is the relative similarity in distributional characteristics of the ATTDRUG variable across outlier detection methods. This would suggest that there were few if any outliers present in the data for this variable. Finally, the full and MGV datasets had kurtosis values that were somewhat larger than those of the other methods included here.

Finally, in order to ascertain how the various outlier detection methods impacted relationships among the variables we estimated correlations for each approach, with results appearing in Table 3. For the full data, correlations among the three variables were all low, with the largest being 0.13. When outliers were detected and removed using *D*^2^, MGV and the three projection methods, the correlations were attenuated even more. In contrast, correlations calculated using the MCD data were uniformly larger than those of any method, except for the value between TIMEDRS and ATTDRUG, which was larger for the MVE data. On the other hand, the correlation between TIMEDRS and ATTHOUSE was smaller for MVE than for any of the other methods used here.

## Discussion

The purpose of this study was to demonstrate seven methods of outlier detection designed especially for multivariate data. These methods were compared based upon distributions of individual variables, and relationships among them. The strategy involved first identification of outlying observations followed by their removal prior to data analysis. A brief summary of results for each methodology appears in Table 4. These results were markedly different across methods, based upon both distributional and correlational measures. Specifically, the MGV and Mahalanobis distance approaches resulted in data that was fairly similar to the full data. In contrast, MCD and MVE both created datasets that were very different, with variables more closely adhering to the normal distribution, and with generally (though not universally) larger relationships among the variables. It is important to note that these latter two methods each removed 230 observations, or nearly half of the data, which may be of some concern in particular applications and which will be discussed in more detail below. Indeed, this issue is not one to be taken lightly. While the MCD/MVE approaches produced datasets that were more in keeping with standard assumptions underlying many statistical procedures (i.e., normality), the representativeness of the sample may be called into question. Therefore, it is important that researchers making use of either of these methods closely investigate whether the resulting sample resembles the population. Indeed, it is possible that identification of such a large proportion of the sample as outliers is really more of an indication that the population is actually bimodal. Such major differences in performance depending upon the methodology used point to the need for researchers to be familiar with the panoply of outlier detection approaches available. This work provides a demonstration of the methods, comparison of their relative performance with a well known dataset, and computer software code so that the reader can use the methods with their own data.

**Table 4:** **Summary of results for outlier detection methods**.

Method	Outliers removed	Impact on distributions	Impact on correlations	Comments
$D_{i}^{2}$	13	Reduced skewness and kurtosis when compared to full data set, but did not fully eliminate them. Reduced variation in TIMEDRS	Comparable correlations to the full dataset	Resulted in a sample with somewhat less skewed and kurtotic variables, though they did remain clearly non-normal in nature. The correlations among the variables remained low, as with the full dataset
MVE	230	Largely eliminated skewness and greatly lowered kurtosis in TIMEDRS. Also reduced kurtosis in ATTHOUSE when compared to full data. Greatly lowered both the mean and standard deviation of TIMEDRS	Resulted in markedly higher correlations for two pairs of variables, than was seen with the other methods, except for MCD	Reduced the sample size substantially, but also yielded variables with distributional characteristics much more favorable to use with common statistical analyses; i.e., very little skewness or kurtosis. In addition, correlation coefficients were generally larger than for the other methods, suggesting greater linearity in relationships among the variables
MCD	230	Very similar pattern to that displayed by MVE	Yielded relatively higher correlation values than any of the other methods, except MVE, and no very low values	Provided a sample with very characteristics to that of MVE
MGV	2	Yielded distributional results very similar to those of the full dataset	Very similar correlation structure as found in the full dataset and for *D*^2^	Identified very few outliers, leading to a sample that did not differ meaningfully from the original
P1	40	Resulted in lower mean, standard deviation, skewness, and kurtosis values for TIMEDRS when compared to the full data, *D*^2^, and MGV, though not when compared to MVE and MCD. Yielded comparable skewness and kurtosis to other methods for ATTDRUG and ATTHOUSE, and somewhat greater variation for these other variables, as well	Very comparable correlation results to the full dataset, as well as *D*^2^ and MGV	Appears to find a “middle ground” between MVE/MCD and *D*^2^/MGV in terms of the number of outliers identified and the resulting impact on variable distributions and correlations
P2	43	Very similar results to P1	Very similar results to P1	Provided a sample yielding essentially the same results as P1
P3	40	Identical results to P1	Identical results to P1	In this case, resulted in an identical sample to that of P1

There is not one universally optimal approach for identifying outliers, as each research problem presents the data analyst with specific challenges and questions that might be best addressed using a method that is not optimal in another scenario. This study helps researchers and data analysts to see the range of possibilities available to them when they must address outliers in their data. In addition, these results illuminate the impact of using the various methods for a representative dataset, while the R code in the Appendix provides the researcher with the software tools necessary to use each technique. A major issue that researchers must consider is the tradeoff between a method with a high breakdown point (i.e., that is impervious to the presence of many outliers) and the desire to retain as much of the data as possible. From this example, it is clear that the methods with the highest breakdown points, MCD/MVE, retained data that more clearly conformed to the normal distribution than did the other approaches, but at the cost of approximately half of the original data. Thus, researchers must consider the purpose of their efforts to detect outliers. If they are seeking a “clean” set of data upon which they can run a variety of analyses with little or no fear of outliers having an impact, then methods with a high breakdown point, such as MCD and MVE are optimal. On the other hand, if an examination of outliers reveals that they are from the population of interest, then a more careful approach to dealing with them is necessary. Removing such outliers could result in a dataset that is more tractable with respect to commonly used parametric statistical analyses but less representative of the general population than is desired. Of course, the converse is also true in that a dataset replete with outliers might produce statistical results that are not generalizable to the population of real interest, when the outlying observations are not part of this population.

### Future research

There are a number of potential areas for future research in the area of outlier detection. Certainly, future work should use these methods with other extant datasets having different characteristics than the one featured here. For example, the current set of data consisted of only three variables. It would be interesting to compare the relative performance of these methods when more variables are present. Similarly, examining them for much smaller groups would also be useful, as the current sample is fairly large when compared to many that appear in social science research. In addition, a simulation study comparing these methods with one another would also be warranted. Specifically, such a study could be based upon the generation of datasets with known outliers and distributional characteristics of the non-outlying cases. The various detection methods could then be used and the resulting retained datasets compared to the known non-outliers in terms of these various characteristics. Such a study would be quite useful in informing researchers regarding approaches that might be optimal in practice.

## Conclusion

This study should prove helpful to those faced with a multivariate outlier problem in their data. Several methods of outlier detection were demonstrated and great differences among them were observed, in terms of the characteristics of the observations retained. These findings make it clear that researchers must be very thoughtful in their treatment of outlying observations. Simply relying on Mahalanobis Distance because it is widely used might well yield statistical results that continue to be influenced by the presence of outliers. Thus, other methods described here should be considered as viable options when multivariate outliers are present. In the final analysis, such an approach must be based on the goals of the data analysis and the study as a whole. The removal of outliers, when done, must be carried out thoughtfully and with purpose so that the resulting dataset is both representative of the population of interest and useful with the appropriate statistical tools to address the research questions.

## Conflict of Interest Statement

The author declares that the research was conducted in the absence of any commercial or financial relationships that could be construed as a potential conflict of interest.

## Appendix

library(MASS)
mahalanobis.out < -mahalanobis(full.data,colMeans(full.data),cov(full.data))
mcd.output < -cov.rob(full.data,method = “mcd,” nsamp = “best”)
mcd.keep < -full.data[mcd.output$best,]
mve.output < -cov.rob(full.data,method = “mve,” nsamp = “best”)
mve.keep < -full.data[mve.output$best,]
mgv.output < -outmgv(full.data,y = NA,outfun = outbox)
mgv.keep < -full.data[mgv.output$keep,]
projection1.output < -outpro(full.data,cop = 2)
projection1.keep < -full.data[projection1.output$keep,]
projection2.output < -outpro(full.data.matrix,cop = 3)
projection2.keep < -full.data[projection2.output$keep,]
projection3.output < -outpro(full.data,cop = 4)
projection3.keep < -full.data[projection3.output$keep,]

*Note that for the projection methods, the center of the distribution may be determined using one of four possible approaches. The choice of method is specified in the **cop** command. For this study, three of these were used, including MCD (**cop = 2**), median of the marginal distributions (**cop = 3**), and MVE (**cop = 4**).

**The functions for obtaining the mahalanobis distance (**mahalanobis**) and MCD/MVE (**cov.rob**) are part of the **MASS** library in R, and will be loaded when it is called. The **outmgv** and **outpro** functions are part of a suite of functions written by Rand Wilcox with information for obtaining them available at his website (http://dornsife.usc.edu/cf/faculty-and-staff/faculty.cfm?pid = 1003819&CFID = 259154&CFTOKEN = 86756889) and his book, *Introduction to Robust Estimation and Hypothesis Testing*.
